# Bioinformatics and Experimental Insights Into miR‐182, hsa_circ_0070269, and circ‐102,166 as Therapeutic Targets for HCV‐Associated HCC

**DOI:** 10.1002/cnr2.70049

**Published:** 2024-12-01

**Authors:** Yasmeen Ishaq, Bisma Rauff, Badr Alzahrani, Aqsa Ikram, Hasnain Javed, Imran Abdullah, Ghulam Mujtaba

**Affiliations:** ^1^ Institute of Molecular Biology and Biotechnology (IMBB) University of Lahore (UOL) Lahore Pakistan; ^2^ Department of Biomedical Engineering UET Lahore Narowal Pakistan; ^3^ Department of Clinical Laboratory Sciences, College of Applied Medical Sciences Jouf University Sakaka Saudi Arabia; ^4^ Provincial Public Health reference lab Lahore Punjab AIDS Control Program Lahore Pakistan; ^5^ Institute of Nuclear Medicine & Oncology (INMOL) Cancer Hospital Lahore Pakistan

**Keywords:** ceRNA, circRNA, HCV induced HCC, hepatocellular carcinoma, miRNA, oncogenic pathways

## Abstract

**Aims:**

Hepatocellular carcinoma (HCC) is a type of malignant tumor and the sixth leading cause of death worldwide. It is caused by HBV, HCV infection, and alcohol consumption. MicroRNAs are typically small, non‐coding RNAs that are involved in the regulation of mRNA expression. Recent studies revealed miRNAs' regulatory roles in liver cancer, linked to risk factors like HCV, HBV infection, alcoholism, drug use, and auto‐immune hepatic disorders. Circular RNAs also belong to the class of non‐coding RNAs; they act as ceRNAs to regulate miRNA expression and regulate different oncogenic pathways in HCC progression. This study aimed to check the hsa_circ_0070269, circ‐102,166 (hsa_circ_0004913), and miR‐182 expression in HCV induced HCC patients.

**Methods:**

Data analysis was used to find out studies related to the role of hsa_circ_0070269, circ‐102,166, and miR‐182 in HCC; miR‐182 targeted genes, their role in different diseases; and miR‐182 interactions with hsa_circ_0070269 and circ‐102,166 in the HCC. It was revealed that the hsa_circ_0070269, circ‐102,166, and miR‐182 correlations in HCV induced HCC have not been explored yet. Therefore, to validate data from literature mining, expression analysis of dysregulated hsa_circ_0070269, circ‐102,166, and miR‐182 was performed in HCV induced HCC patients using RT‐PCR.

**Results:**

It was found that miR‐182 was significantly upregulated and acts as an oncomiRNA in HCV induced HCC, and hsa_circ_0070269 and circ‐102,166 were downregulated in HCV induced HCC. We have identified that miR‐182 relative expression level was significantly high (*p* < 0.0029), while has_circ_0070269 (*p* < 0.002) and circ‐102,166 (*p* < 0.002) were significantly downregulated in HCV‐HCC patients as compared to expression in healthy individuals.

**Conclusion:**

Our data revealed that miR‐182 acts as an oncomiRNA in HCC development. Hsa_circ_0070269 and circ‐102,166 are highly expressed in healthy controls compared to HCV induced HCC patients, can sponge miR‐182 expression by acting as tumor suppressors, and can be used as biomarkers and targets for HCC treatment.

**Abbreviations**
Hepatocellular carcinoma: HCC
Hepatitis B virus: HBV
Hepatitis C virus: HCV
Chronic hepatitis C: CHC
α‐fetoprotein: AFP
microRNAs: miRNAs
3′ untranslated region: 3′UTR
Non‐small cell lung cancer: NSCLC
Glioblastoma multiforme: GBM
Forkhead Box O3: FOXO3
Circular RNAs: circRNAs
Competing endogenous RNA: ceRNA
Interferon alpha: IFN‐α
World Health Organization: WHO
Circular RNAs: circRNAs
Competing endogenous RNA: ceRNA
Osteosarcoma: OS
StAR‐related lipid transfer domain protein 13: STARD13
Gene Expression Omnibus: GEO
Messenger RNA: mRNA
Magnetic resonance imaging: MRI
Computed tomography scan: CT
Ethylenediaminetetraacetic acid: EDTA
Kyoto Encyclopedia of Genes and Genomes: KEGG
Gene Ontology: GO
Missing in Metastasis: MIM
Ras homolog family member A: Rho A
Hypoxia‐inducible factor 1α: HIF1α
Prolyl hydroxylase domain enzymes: PHD
F‐Box and WD Repeat Domain Containing 7: FBXW7
F‐Box and WD Repeat Domain Containing11: FBXW11
Forkhead Box O3: FOXO3
High‐grade serous ovarian carcinoma: HG‐SOC
Breast Cancer Gene 1: BRCA1
High Mobility Group AT‐Hook 2: HMGA2
Metastasis Suppressor 1: MTSS1
Colon rectal cancer: CRC
Anti‐angiogenic factor thrombospondin‐1: TSP‐1
Insulin‐like growth factor 1 receptor: IGF1R
Angiopoietin Like 1: ANGPTL1
Ras p21 protein activator 1: RASA1
Programmed cell death protein 4: PDCD4
SRY‐related HMG box 11: SOX11
CCAAT enhancer binding protein: Cebpa
Neuronal pentraxin: NPTX1
Melanocyte‐inducing transcription factor: MITF
E2F transcription factor 1: E2F1
Solute carrier family 7 membrane 11: SLC7A11
MAF BZIP transcription Factor F: MafF

## 1 Introduction

Hepatocellular carcinoma (HCC) is a very common type of solid tumor [1]. It is the 6th main cause of mortality globally [2]. Its incidence rates have increased in the US since the mid‐1970s [3]. It is estimated that about 5–10 million new cases are reported annually [4, 5, 6]. It is mainly caused by hepatitis C (HCV), hepatitis B (HBV) infection, and alcoholism [7, 8, 9]. The prognosis of HCC is mostly related to the cancer stage; unfortunately, most HCC cases are diagnosed at the last stage due to the current screening methods' low efficacy. For instance, α‐fetoprotein (AFP) screening has low diagnostic capacity, particularly in cases of liver cirrhosis [10]. In the past few years, a huge amount of studies have been published on the miRNAs’ regulatory roles in liver cancer, and they are associated [11, 12] with different risk factors like HCV, HBV infection, alcohol consumption, drug toxicity, and auto‐immune hepatic disorders [13, 14].

HCV is a main risk factor for HCC. It is a positive‐sense, enveloped, and single stranded RNA virus. It belongs to the *Flaviviridae* family [15]. The diameter of HCV particles is about 68 nm [16], and its genome consists of 9600 nucleotides that encode a single polyprotein consisting of ~3000 amino acids [17]. Over 170 million individuals worldwide were infected by HCV, and almost 70 million were chronically infected [15]. HCV causes an acute infection that commonly resolves within 6 months of exposure [18]. When acute phase infection does not clear instinctively, it develops into chronic hepatitis C (CHC). Usually, CHC has a slow rate [19]. Vitamin D is an immune modulator and is mostly deficient in CHC patients. The role of vitamin D in response to interferon alpha (IFN‐α) based therapy [8]. Administration of vitamin E seems to help reduce proinflammatory indicators [20]. Characteristics of CHC include constant hepatic inflammation, the development of hepatic fibrosis, and cirrhosis [19]. WHO eradication goals require in‐depth and analytical HCV characterization epidemiology at regional and national levels to evolve cheap, targeted prevention and treatment interventions [21]. Different factors have been investigated in the treatment and pathology of HCV induced HCC including microRNAs (miRNAs) [22].

miRNAs are typically small non‐coding RNAs, comprising 20–22 nucleotides and accounting for around 1%–2% of genes in mammals [23]. They play a main role in the regulation of mRNA expression by binding to the 3′UTR (untranslated region) of the target gene [24]. They can act as reputed tumor suppressor genes, causing cell cycle halts, inducing apoptosis, and decreasing tumor metastasis and angiogenesis by hindering invasion and migration [25]. MiR‐182 belongs to the miR‐183 family and is found on chromosome 7q31‐34. It was reported that miR‐182 is involved in HCV replication and pathogenesis [26]. Several research studies have described that miR‐182 could act as both an oncomiRNA and a tumor suppressor miRNA, representing its dual role in cancer. miR‐182 dysregulation is involved in different diseases [27], like HCC [28], prostate cancer [29], breast cancer [30], non‐small cell lung cancer (NSCLC) [31], and glioblastoma multiforme (GBM) [32].

In addition, circular RNAs (circRNAs) are non‐coding RNAs. It has 5′ and 3′ free ends that are joined together by a covalent bond and make a closed‐loop structure [33]. It has the ability to control miRNA expression by acting as a ceRNA (competing endogenous RNA), and circRNA can bind with miRNA and sponge their expression, thus indirectly involved in gene regulation [34, 35]. Instinctively, circRNAs have a vital regulatory role in the pathological and biological mechanisms of several illnesses [36], including HCC; however, different unidentified circRNAs and their targeted genes still require study [37]. CircRNAs have the ability to interact with miRNAs by sponging, upregulating, and downregulating them. In most cases, high expression of circRNAs sequesters the miRNAs that de‐repress mRNA translation, which makes oncogenic proteins [38]. MiR‐182 acts as a tumor suppressor and downregulation inhibits cell proliferation and induces apoptosis in cervical cancer and osteosarcoma (OS) [39]. MiR‐182‐5p acts as an oncogene, highly expressed and involved in HCC progression. The circ_0003570 regulates StAR‐related lipid transfer domain protein 13 (STARD13) sponge miR‐182‐5p expression and inhibits HCC progression [40]. It has been reported that hsa_circ_0070269 and circ‐102,166 (hsa_circ_0004913) act as tumor suppressor genes in liver tumorigenesis [22]. Hsa_circ_0070269 binds with miR‐182 and upregulates the expression level of their downstream target neuronal pentraxin 1 (NPTX1). Therefore, hsa_circ_0070269 can inhibit proliferation and invasion in HCC cells by sponging miR‐182 expression [41]. Circ‐102,166 competitively binds and can sponge miR‐182 and control the expression of their different downstream targets, including metastasis suppressor 1 (MTSS1), SRY‐Box Transcription Factor 7 (SOX7), Forkhead box O3a (FOXO3a), and cellular Myc (c‐Myc). So, Circ‐102,166 can sponge miR‐182 and reduce the growth, migration, invasion, and tumorigenicity in HCC cells. However, the mechanism behind the miR‐182 dysregulation in HCC remains unclear [42].

It is important to mention that previous studies reported the involvement of hsa_circ_0070269 and circ‐102,166 in HCC, but did not mention the risk factor of HCC development, and no study reported the interaction of miR‐182 with hsa_circ_0070269 and circ‐102,166 in HCC caused by HCV infection. In the present, we screened out research studies that reported the role of miR‐182, has_circ_0070269, circ‐102,166 role in HCC development; the role of miR‐182 in different diseases and HCC; we retrieved data reporting the miR‐182 interactions with has_circ_0070269, circ‐102,166 in HCC through literature mining and bioinformatics analysis. Only the individual interaction of has_circ_0070269 miR‐182; and circ‐102,166 with miR‐182 in HCC development has been evident in a few studies. Therefore, to validate the role of selected circRNAs and miRNA in HCV induced HCC patients, the expression profiling analysis of has_circ_0070269, circ‐102,166, and miR‐182 was performed.

The present study aims to screen out miR‐182 expression in different diseases and HCC development through literature mining; to screen out hsa_circ_0070269 and circ‐102,166 reported in HCC through literature mining and bioinformatics analysis; to find out the expression profile of hsa_circ_0070269 and circ‐102,166 in comparison with miR‐182 in HCV induced HCC patients.

Currently, we have reviewed research studies focusing on the role of has_circ_0070269 and circ‐102,166 in HCC development, as well as the involvement of miR‐182 in various diseases and HCC. The data retrieved highlighted interactions between miR‐182 and has_circ_0070269, circ‐102,166 in HCC through literature mining and bioinformatics analysis. However, only a few studies have examined the individual interactions of has_circ_0070269 with miR‐182, and circ‐102,166 with miR‐182 in the context of HCC development [22]. To further investigate the roles of these circRNAs and miRNA in HCV induced HCC patients, we performed expression profiling analysis of has_circ_0070269, circ‐102,166, and miR‐182. The objectives of this study are to: (1) examine the expression of miR‐182 in various diseases and its role in HCC development through literature mining; (2) identify the role of has_circ_0070269 and circ‐102,166 in HCC through literature mining and bioinformatics analysis; and (3) compare the expression profiles of has_circ_0070269 and circ‐102,166 with miR‐182 in HCV induced HCC patients.

## 2 Methods

### 2.1 Data Mining

This research study was conducted to evaluate miR‐182, hsa_circ_0070269, and circ‐102,166 expression levels, based on the data analysis approach that was already reported in our previous study [22]. We screened out studies that reported the role of hsa_circ_0070269 and circ‐102,166 in HCC through data mining. We also screened out all possible studies that reported the miR‐182 role in different diseases and HCC development through literature mining. Moreover, we screened out all possible studies based on miR‐182 interactions with hsa_circ_0070269, circ‐102, and circ‐166 in HCC development. The present research was designed to identify the correlation of miR‐182 with has_circ_0070269 and circ‐102,166 in HCV induced HCC.

### 2.2 Bioinformatics Analysis

**FIGURE 1:**
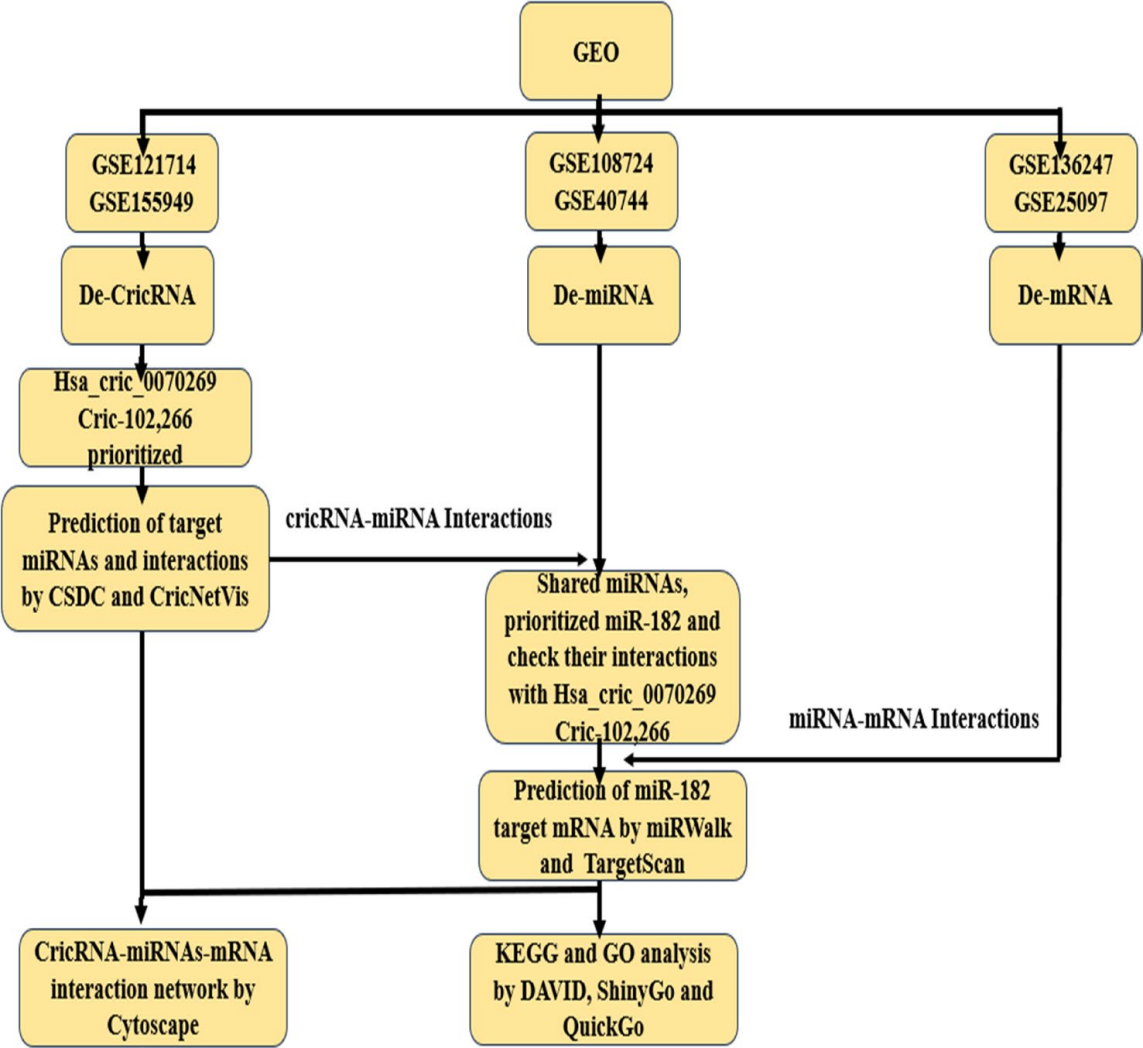
Flow chart of bioinformatic analysis.

Different database and online bioinformtic tools were used in bioinfromatic analysis as presented in Figure 1.

### 2.3 Data Collection and Processing

In the present study, differentially expressed circRNAs, miRNA, and mRNA were identified using Gene Expression Omnibus (GEO) [43] (https://www.ncbi.nlm.nih.gov/geo/) (access date: 15 July 2023). Two circRNAs’ datasets, GSE121714 and GSE155949; two miRNAs’ datasets, GSE40744 and GSE108724; and two mRNAs’ datasets, GSE136247 and GSE25097, were retrieved. Fu et al., dataset GSE121714 included 30 samples (10 HCC with metastasis, 10 HCC without metastasis, and 10 controls). Han et al., dataset GSE155949 included 98 samples (49 HCC and 49 controls). Diaz et al., dataset GSE40744 included 76 samples (9 HCC, 17 HCV‐associated cirrhosis surrounding HCC, 18 HCV‐associated cirrhosis without HCC, 13 HBV‐associated acute liver failure, 7 hepatic resections for liver angioma, and 7 controls). Zhu et al., datasets GSE108724 included 14 samples (7 HCC and 7 normal tissue adjacent to HCC). Cerapio et al. dataset GSE136247 included 69 samples (39 HCC and 30 controls). Chunsheng et al. dataset GSE25097 included 557 samples (40 cirrhotic, 243 non‐tumor liver, 268 HCC, and 6 controls).

### 2.4 CircRNA, miRNA and mRNA Target Prediction

We prioritized has_circ_0070269 and circ‐102,166 for further analysis because very limited data exists on their circRNAs. We used three different bioinformatic databases, like CSCD [44] (http://gb.whu.edu.cn/CSCD/), CircNetVis [45] (https://www.meb.ki.se/shiny/truvu/CircNetVis/), circBase [46] (http://www.circbase.org/), to predict circRNA target miRNAs.

In the miRNA target partition of has_circ_0070269 and circ‐102,166, we prioritized miR‐182 for further analysis. We used miRWalk 3.0 [47] (http://mirwalk.umm.uni‐heidelberg.de/), miRBase [48] (https://www.mirbase.org/) and TargetScan 8.0 [49] (https://www.targetscan.org/vert_80/) for miR‐182 target prediction.

### 2.5 CircRNA–miRNA–mRNA Network Construction

For circRNA‐miRNA‐mRNA regulatory network construction, we used the cytoHubba [50] a plugin in the Cytoscape 3.10.2 software.

### 2.6 KEGG Pathway and GO Analysis

The QuickGO [51] (https://www.ebi.ac.uk/QuickGO/annotations?geneProductId=URS00001CC379_9606), the Database for Annotation, Visualization and Integrated Discovery [52] (DAVID; https://david.ncifcrf.gov/tools.jsp), and ShinyGO 0.77 [53] (http://bioinformatics.sdstate.edu/go77/) were used to perform Kyoto Encyclopedia of Genes and Genomes (KEGG) pathway and gene ontology (GO) analysis.

### 2.7 Experimental Analysis

Based on the above data analysis, we checked the miR‐182, hsa_circ_0070269, and circ_102,166 expression levels in HCV‐HCC patients as well as in healthy control by adopting the following steps:

### 2.8 Sample Collection

Twenty healthy volunteers and 60 HCV induced HCC patients from different cancer hospitals in Pakistan and 20 healthy individuals as normal controlswere included in this study. The HCV induced HCC group included 42 men and 18 women with an age range of 30–80 years (*M* = 55.25, SD = 9.205). Furthermore, the 20 healthy control groups included 8 men and 12 women, with an age range of 19–60 years (*M* = 34.40, SD = 16.7), as mentioned in Table 1.

**TABLE 1:** Patients and healthy controls involved in HCV induced HCC study.

Variables	HCC patients (*n* = 60)	Healthy control (*n* = 20)	*p*
Age			0.02
< 50	14 (23.33%)	7 (35.00%)	
≥ 50	46 (76.67%)	15 (65.00%)	
Gender			0.00
Male	42 (70.00%)	8 (40.00%)	
Female	18 (30.00%)	12 (60.00%)	

*Note: p*‐value by *t*‐test for continuous variables and Chi2 test for binary/categorical variables.

### 2.9 Inclusion and Exclusion Criteria

In the present study, inclusion and exclusion criteria were adopted. Inclusion criteria include: (i) Patients diagnosed with HCV induced HCC by at least two imaging techniques (i.e., hepatic ultrasound along with MRI or CT, or both); (ii) patients with an age above 18 years old; and (iii) patients with advanced stage HCC.

Exclusion criteria include: (i) Patients with a history of other than HCV infection or HCC induced by HBV or alcoholism; (ii) patients with a history of other types of cancer.

All HCV induced HCC patients included in this research study were at the last stage of cancer and had received no other treatment before the sample collection. Informed consent was taken from all participants. A 5 ml peripheral blood sample was collected in EDTA vacutainers and stored at −80°C. This research study was approved by the Research Ethics Committee of the University of Lahore (REC‐UOL).

### 2.10 HCV Detection

HCV detection in serum was performed on the cobas 6800 system (Roche Diagnostics, Mannheim, Germany) by using COBAS AmpliPrep/COBAS TaqMan HCV Quantitative Tests kit.

### 2.11 Total RNA Extraction

Total RNA was extracted from whole blood samples using Quick‐RNA MiniPrep Plus (ZYMO RESEARCH Cat # R1057, USA), and its quantity and quality were calculated with the help of the Qubit RNA HS Assay Kit (Catalog # Q32852) on the Qubit 4 Fluorometer (Thermo Fisher Scientific, USA).

### 2.12 RT‐PCR

Expression of dysregulated miR‐182, hsa_circ_0070269, and circ‐102,166 was checked in blood samples of 60 HCV induced HCC patients and 20 healthy individuals (as proper control) using RT‐PCR. Total RNA was used for cDNA synthesis with the RevertAid First Strand cDNA Synthesis Kit (Thermo Scientific #K1622). For RT‐PCR, First Strand cDNA (2 ml ) was used with Maxima SYBR Green/ROX qPCR Master Mix (2×) (Thermo Scientific #K0221). Glyceraldehyde‐3‐phosphate dehydrogenase (GAPDH) was used as an internal reference gene. Relative expression levels of miR‐182, hsa_circ_0070269, and circ‐102,166 were calculated with the help of the ^ΔΔ^CT method [54]. Primer sequences are mentioned in Table 2.

**TABLE 2:** Primer sequences.

Non‐coding RNAs	Nucleotide sequence 5'–3'
miR‐182	F‐CCCGTTTTTGGCAATGGTAGAA
	R‐GCCCCATAGTTGGCAAGTCTA
Hsa_circ_0070269	F‐ACATTTTGTTTCCCGTGCCT
	R‐TACGCATGGCTCTCCTTCTG
Circ‐102,166	F‐GCACACAGCAGACACAACAG
	R‐ACACGCTGTAGTCGAGAAGC
GAPDH	F‐CGACCACTTTGTCAAGCTCA
	R‐AGG GGT CTA CAT GGC AAC TG

### 2.13 Statistical Analysis

To validate the relative expression of miR‐182, hsa_circ_0070269, and circ‐102,166 in blood samples of HCV‐HCC patients and the normal controls (3 replicates of each sample), RT‐PCR was performed. To find out significant differences in the miR‐182, circ‐102,166, and hsa‐circ‐0070269 expressions between the HCV‐HCC patients and healthy individuals, a Student's *t*‐test and One‐way ANOVA were used to perform statistical analysis on GraphPad Prism Version 8.0.2.

## 3 Results

### 3.1 Data Mining

In the present study, we screened out all research papers that reported the miR‐182 hsa_circ_0070269 and circ‐102,166 role in various diseases and HCC development through literature mining. We have found that only few studies reported the role of hsa_circ_0070269 and circ‐102,166 in HCC development.

### 3.2 Bioinformatics Analysis

#### 3.2.1 Differentially Expressed circRNAs‐miRNAs‐mRNAs

**FIGURE 2:**
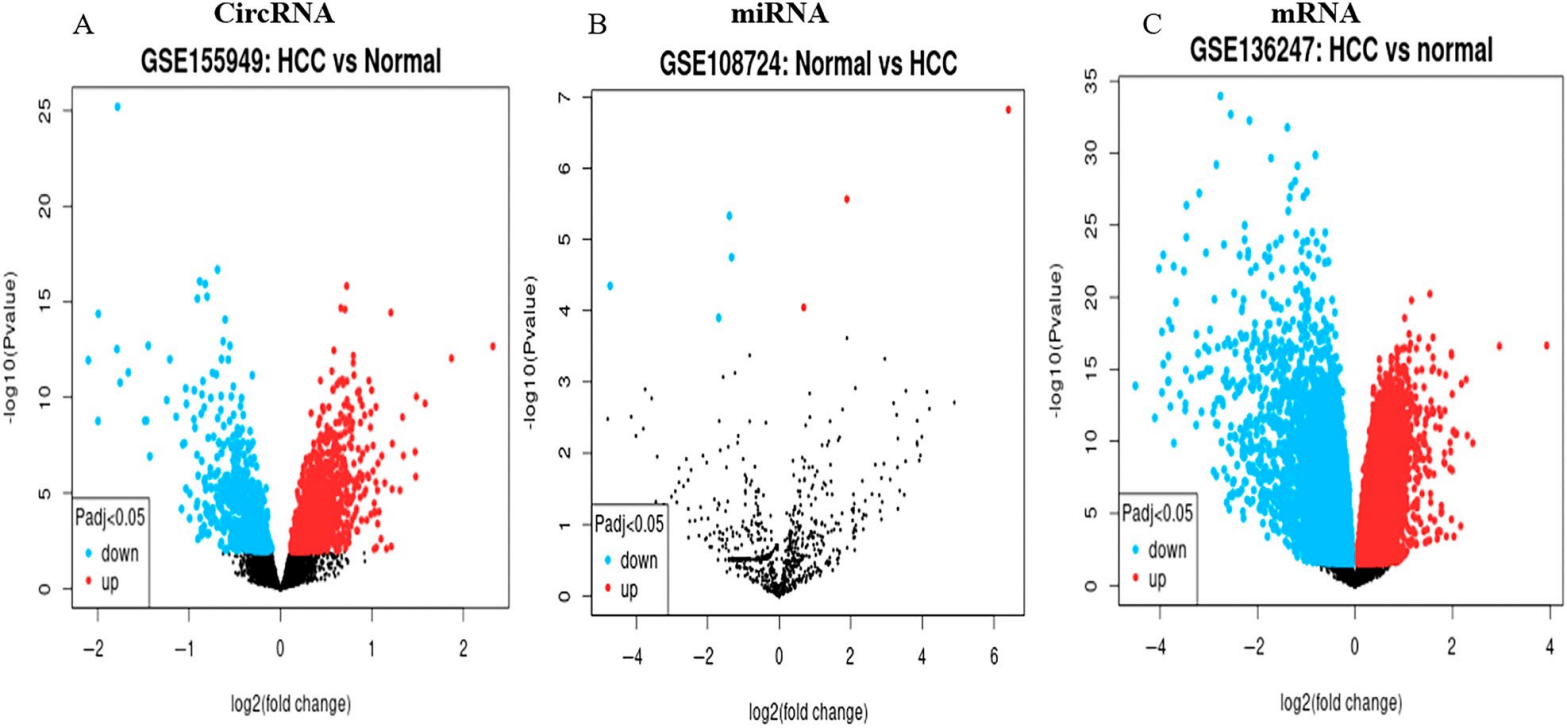
Volcano plots of differentially expressed circRNA, miRNA and mRNA in HCC. (A) Dysregulated circRNA. (B) Dysregulated miRNA. (C) Dysregulated mRNA. Blue color indicates expression downregulation; red color indicates expression upregulation.

We used GEO2R (https://www.ncbi.nlm.nih.gov/geo/geo2r/) tool to create differentially expressed circRNAs, miRNAs and mRNA profiles. We have found dysregulated circRNAs, miRNAs and mRNA related HCC (Figure 2).

### 3.3 CircRNA, miRNA and mRNA Interaction Identification

**FIGURE 3:**
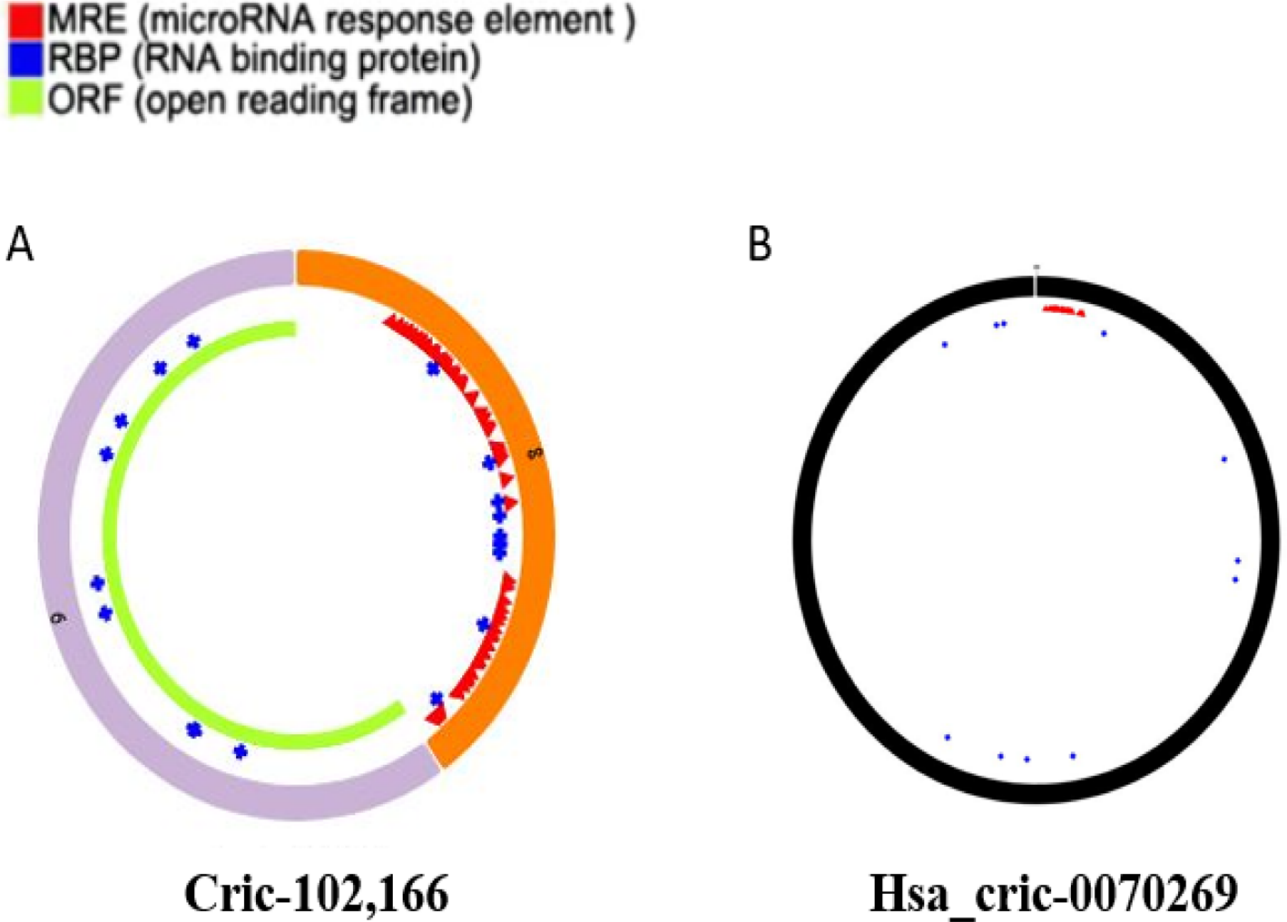
Structural pattern of circRNAs: (A) circ‐102,166; (B) hsa_circ‐0070269.

From GEO analysis, we have prioritized hsa_circ_0070269 and circ‐102,166 for further analysis because we found very limited data related to hsa_circ_0070269 and circ‐102,166. The CSCD database [44] was used to find out the basic structural patterns of selected the circRNAs mentioned in Figure 3.

A growing body of evidence indicates that some circRNAs are involved in tumor development by acting as "decoys" to sequester miRNA expression. To depict whether these circRNAs play the same role in HCC, we used the online databases CSCD [44] and CircNetVis [45] for the collection of putative target miRNAs of hsa_circ_0070269 and circ‐102,166. We have identified a large number of miRNA targets for hsa_circ_0070269 and circ‐102,166; among them, we prioritized miR‐182 for further analysis. We used miRWalk [47] and TargetScan [49] for the identification of miR‐182 targets. We find 1330 target genes for miR‐182.

### 3.4 CircRNA–miRNA–mRNA Network Construction

**FIGURE 4:**
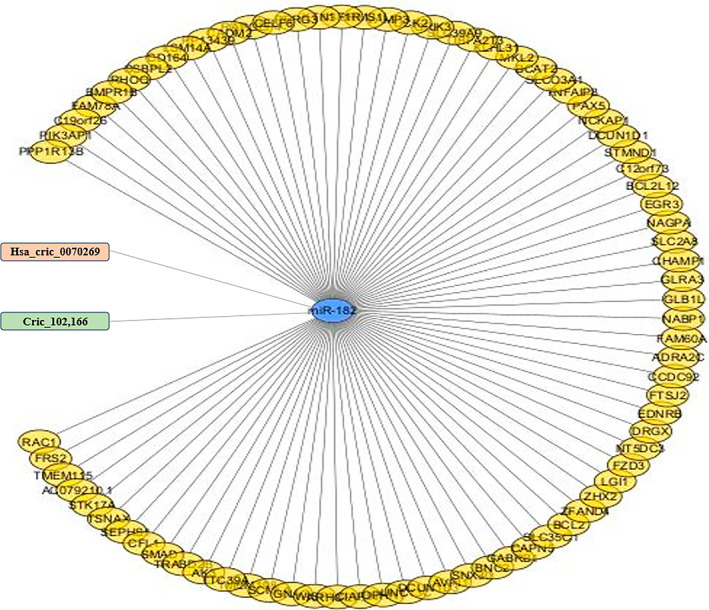
CircRNA‐miRNA‐mRNA network construction.

The circRNA‐miRNA‐mRNA regulatory network of circ‐102,166 and hsa_circ_0070269 was constructed by Cytoscape 3.10.2 software. The network circ‐102,166 and hsa_circ_0070269 included miR‐182 forming interaction with their target genes (Figure 4).

### 3.5 KEGG Pathway and GO Analysis

**FIGURE 5:**
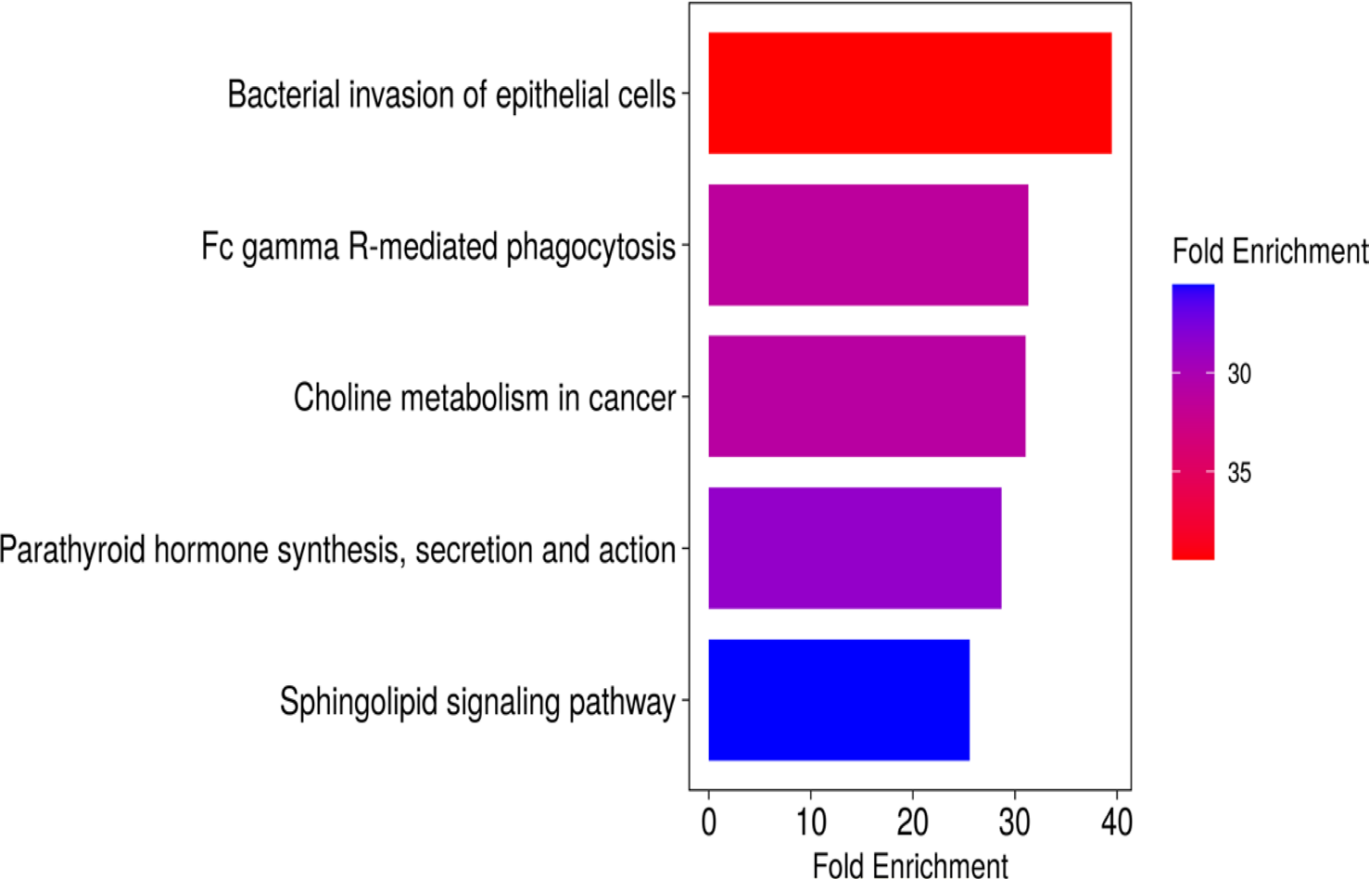
KEGG pathway analysis: (A) Bacterial invasion of epithelial cells; (B) Fc gamma R‐mediated phagocytosis; (C) choline metabolism in cancer; (D) parathyroid hormone synthesis, secretion, and action; and (F) Sphingolipid signaling pathway. The bar represents the −log10 (*p*‐value).

DAVID [52] and QuickGO [51] were used to find out gene annotation. We performed a KEGG pathway enrichment analysis with the help of ShinyGO 0.77 [53] to better understand the function of the differentially expressed genes. The top 10 significant KEGG pathways and their functional classification by 5 main categories include bacterial invasion of epithelial cells; Fc gamma R‐mediated phagocytosis; choline metabolism in cancer; parathyroid hormone synthesis, secretion and action; and Sphingolipid signaling pathway (Figure 5).

### 3.6 miR‐182 Role in Different Diseases

**FIGURE 6:**
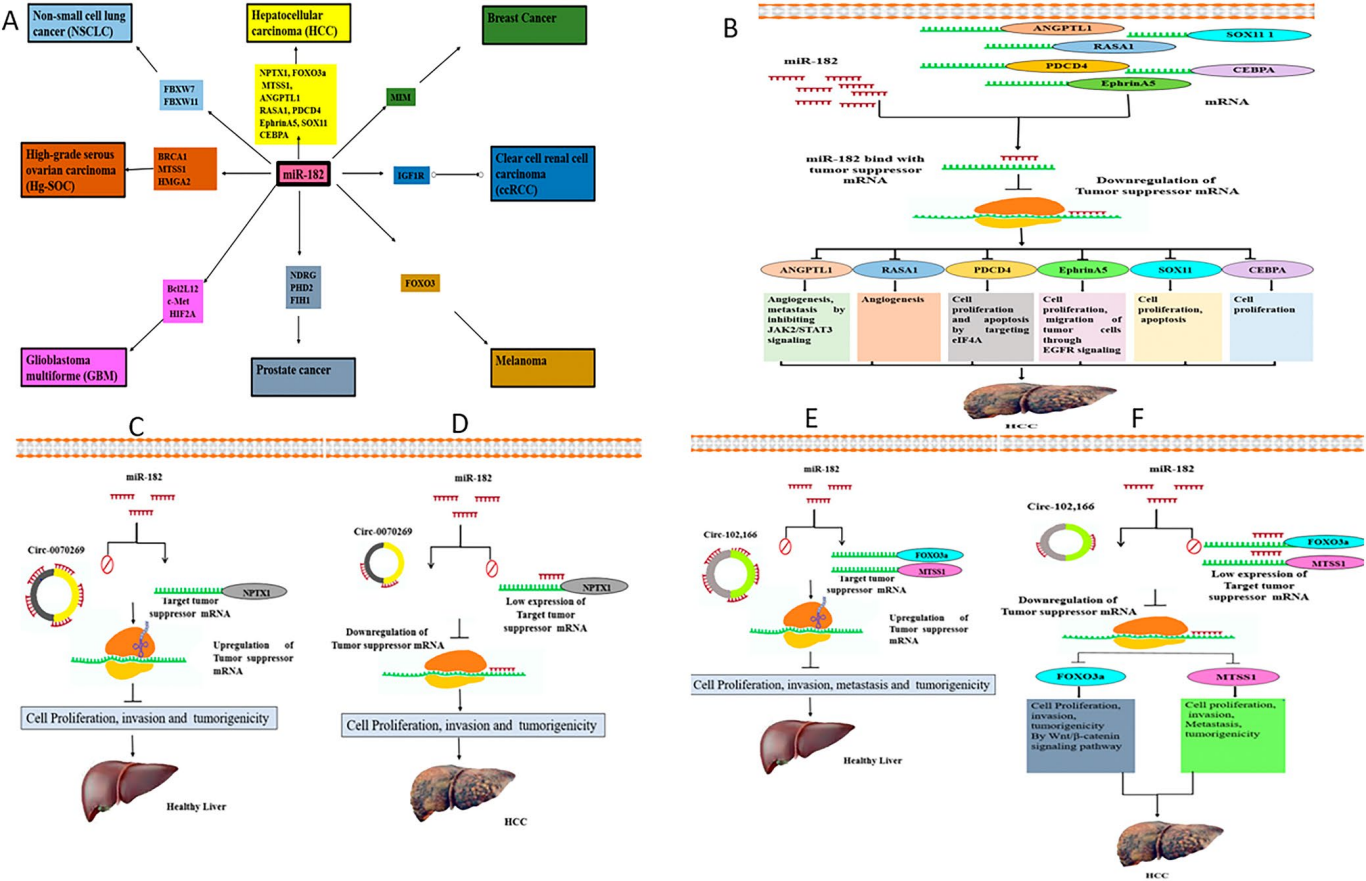
miRNAs and circRNAs role in pathogenesis. (A) miR‐182 role in different diseases by targeting different pathways. (B) miR‐182 role in HCC development by targeting tumor suppressor genes. (C) Hsa_circ_0070269 and miR‐182 in healthy liver. (D) Hsa_circ_0070269 and miR‐182 interaction HCC progression. (E) Circ‐102,166 and miR‐182 interaction in the healthy liver. (F) Circ‐102,166 and miR‐182 interaction in HCC progression.

Several studies reported that miR‐182 could act as both an oncomiRNA and a tumor suppressor miRNA, representing its dual role in cancer. miR‐182 dysregulation is involved in different diseases [27], like HCC [28], breast cancer [30], NSCLC [31], prostate cancer [29], and GBM [32], as presented in Figure 1. In breast cancer, high expression of miR‐182 suppresses the MIM (Missing in Metastasis) and triggers Ras homolog family member A (Rho A), which induces metastasis [30]. In prostate cancer, miR‐182 upregulation activates hypoxia‐inducible factor 1α (HIF1α) signaling by targeting and inhibiting HIF‐1 (FIH1) and prolyl hydroxylase domain enzymes (PHD) [27]; it also induces cell proliferation and invasion by targeting the c‐myc downstream‐regulated gene 1 (NDRG1) [29]. In NSCLC, miR‐182 induces cell growth by inhibiting of FBXW7 and FBXW11 (F‐Box and WD Repeat Domain Containing 7 and 11) [55]. In GBM, miR‐182 downregulation contributes to cancer [32]. In melanoma, miR‐182 upregulation induces metastasis by targeting Forkhead Box O3 (FOXO3) [56]. In high‐grade serous ovarian carcinoma (HG‐SOC), high expression of miR‐182 promotes tumorigenesis by targeting Breast Cancer Gene 1 (BRCA1), High Mobility Group AT‐Hook 2 (HMGA2), and Metastasis Suppressor 1 (MTSS1) genes [57]. In colon rectal cancer (CRC), miR‐182 induces angiogenesis by targeting the anti‐angiogenic factor thrombospondin‐1 (TSP‐1) [58]. In clear cell renal cell carcinoma (ccRCC), miR‐182 is downregulated, and their target insulin‐like growth factor 1 receptor (IGF1R) is highly expressed and induces invasion and migration of cancer cells [59], as mentioned in Figure 6A (Table S1).

### 3.7 miR‐182 Role in HCC Development

We performed bioinformatic analysis to find out all possible miR‐182 target genes, which are important key players in oncogenic pathways involved in different pathological conditions as well as in HCC development.

Extensive investigations on the function of miR‐182 in HCC have exposed that miR‐182 is oncomiRNA and highly expressed in HCC [60]. In HCC cells, miR‐182 targets Angiopoietin Like 1 (ANGPTL1) and enhances angiogenesis and metastasis by JAK2/STAT3 signaling pathways [60]; it induces metastasis by targeting MTSS1 [28]; it induces angiogenesis by targeting Ras p21 protein activator 1 (RASA1) [61]; it promotes migration of cells by targeting the programmed cell death protein 4 (PDCD4) [62]; it promotes proliferation and invasion by targeting EphrinA5 [63]; it inhibits apoptosis and induces cell proliferation by targeting SRY‐related HMG box 11 (SOX11); and it induces cell proliferation by targeting the CCAAT enhancer binding protein (Cebpa) gene [64].

miR‐182 binds with tumor suppressor genes like ANGPTL1, MTSS1, RASA1, PDCD4, EphrinA5, SOX11, and Cebpa and suppresses their expression, which leads to HCC development, as represented in Figure 6B (Table S1).

### 3.8 Hsa_circ_0070269 Role HCC

Very few studies have reported the hsa_circ_0070269 role in HCC development. It has been reported that hsa_circ_0070269 acts as a tumor suppressor in HCC development. It is downregulated in HCC [22]. Su and colleagues observed that miR‐182 acts as an oncomiRNA in HCC progression and targets neuronal pentraxin (NPTX1). Hsa_circ_0070269 can inhibit the cell growth and invasion of HCC by sponging the miR‐182 expression and elevating the expression of NPTX1.

Hsa_circ_0070269 upregulation decreases cell growth, invasion, and tumorigenicity of HCC by sequestering the expression of miR‐182 and increasing the expression of NPTX1 in healthy controls, as presented in Figure 6C.

When hsa_circ_0070269 is downregulated, miR‐182 binds with the 3′UTR of NPTX1 and enhances cell growth, invasion, and tumorigenicity of HCC, as displayed in Figure 6D (Table S2).

### 3.9 Circ‐102,166 Role in HCC

A very limited number of studies related to circ‐102,166 role in HCC development are present. It has been reported that the role of circ‐102,166 as a tumor suppressor gene in the development of HCC [22]. Circ‐102,166 is downregulated in HCC patients and can sponge miR‐182 expression and reduce the cell growth, migration, invasion, and tumorigenicity of HCC by upregulating the miR‐182 targets’ MTSS1 and FOXO3a [42].

High expression of circ‐102,166 by sponging miR‐182 expression and upregulating MTSS1 and FOXO3a in healthy liver, as presented in Figure 6E.

When the circ‐102,166 expression is low, miR‐182 targets MTSS1, and FOXO3a inhibits their expression and promotes cell growth, migration, invasion, and tumorigenicity of HCC, as displayed in Figure 6F (Table S3).

### 3.10 Experimental Analysis

#### 3.10.1 Expression Analysis of miR‐182

**FIGURE 7:**
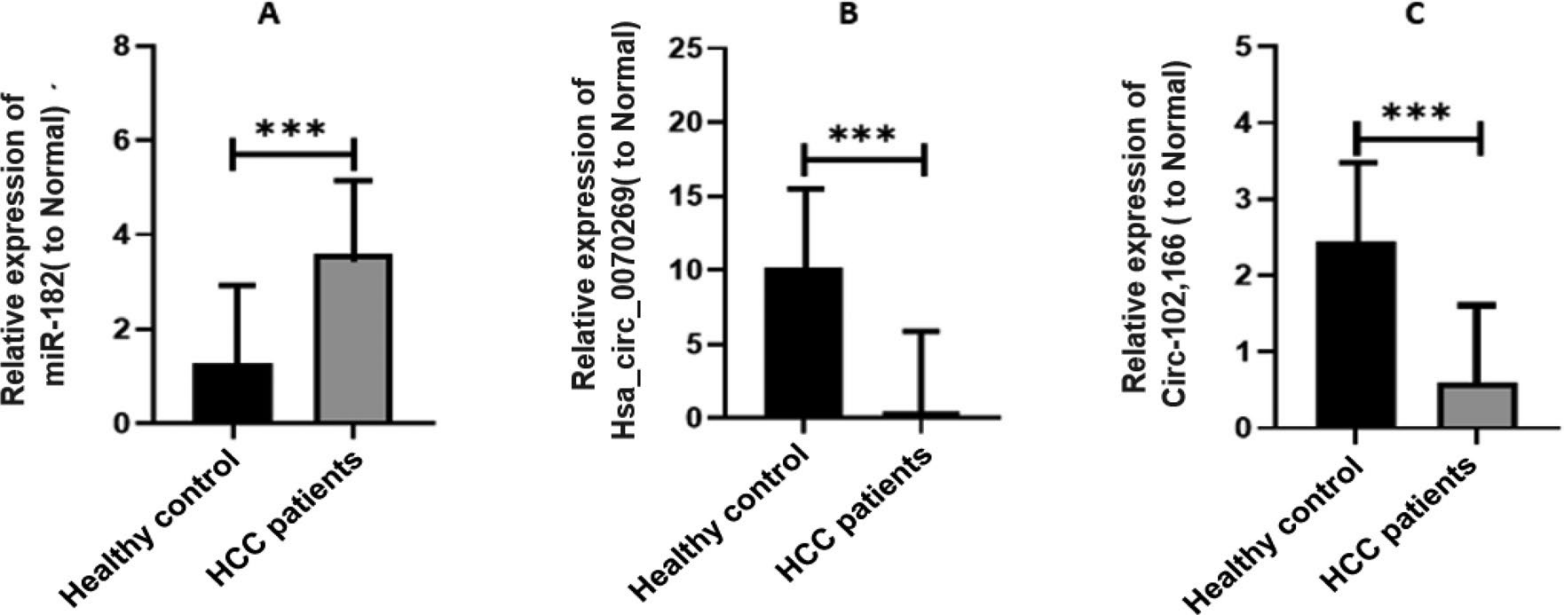
Expression analysis of miR‐182, hsa_circ_0070269, and circ‐102,166. (A) MiR‐182 expression level in the blood samples of HCV‐HCC patients and healthy controls. (B) Hsa_circ_0070269 expression level in blood samples of HCV‐HCC patients and healthy individuals. (C) Circ‐102,166 expression level in blood samples of HCV‐HCC patients and healthy controls.

miR‐182 acts as an oncomiRNA in HCC. In the current study, we performed expression profiling analysis of the miR‐182 in HCV‐HCC patients. We detected that miR‐182 was upregulated (**p < 0.0029) in HCV‐HCC patients as compared to healthy individuals, as mentioned in Figure 7A.

#### 3.10.2 Expression Analysis of hsa_circ‐0070269

Hsa_circ‐0070269 expression analysis in the HCV‐HCC group and control group revealed that hsa_circ‐0070269 expression was significantly low (***p < 0.002) in HCV‐HCC patients as compared to controls, as mentioned in Figure 7B.

#### 3.10.3 Expression Analysis of circ‐102,166

The circ‐102,166 expression analysis among the HCV‐HCC and the control groups. We observed that circ‐102,166 was significantly low (***p < 0.002) in the HCV‐HCC patient group as compared to the control group, as displayed in Figure 7C.

### 3.11 Comparison of the Relative Expression of miR‐182 and hsa_circ_0070269

**FIGURE 8:**
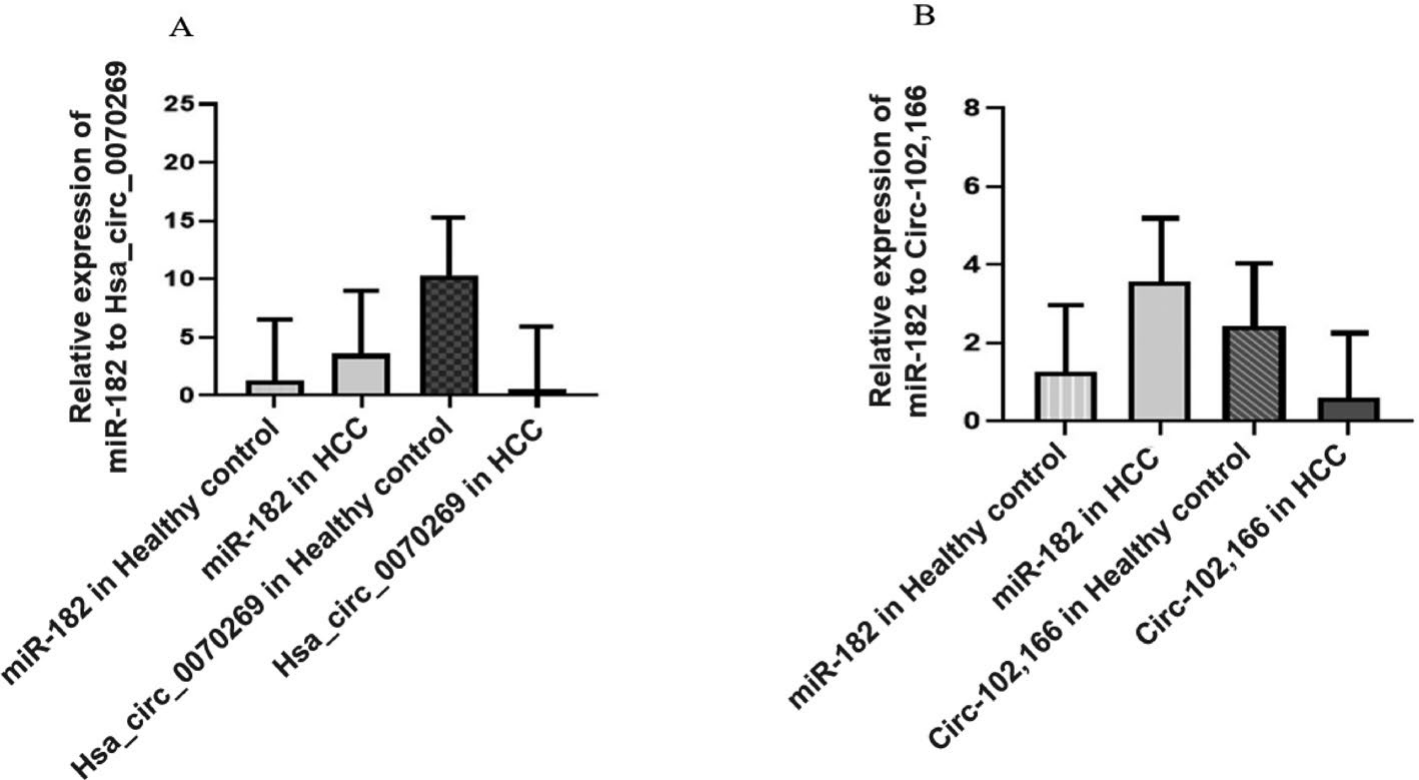
Comparison of the relative expression of the non‐coding RNA in HCV induced HCC and healthy controls. (A) Comparative relative expression of hsa_circ_0070269 and miR‐182. (B) Comparative relative expression of circ‐102,166 and miR‐182.

We checked the miR‐182 and hsa_circ_0070269 expression from blood samples of HCV induced HCC patients; hsa_circ‐0070269 was significantly downregulated and miR‐182 was significantly expressed (***p < 0.0001) in the HCV induced HCC patients, as displayed in the Figure 8A.

### 3.12 Comparison of the Relative Expression of miR‐182 and circ‐102,166

Real‐time PCR amplification was used to find out the miR‐182 and circ‐102,166 expression levels in the blood samples of HCV induced HCC patients. Moreover, we discover that the HCV induced patients have downregulated expression of circ‐102,166 and elevated expression of miR‐182 (***p < 0.0008), as presented in Figure 8B.

## 4 Discussion

HCV is a positive‐strand RNA virus that is one of the causative agents of chronic hepatitis infection, cirrhosis, and HCC [8]. HCV‐HCC development is a process consisting of multi‐step that may take 20–40 years to complete [65]. After the acute phase of infection, the condition abruptly resolved in 18%–34% of HCV‐infected individuals. The condition is termed CHC if the acute HCV infection is not resolved [19]. HCV infection progresses to chronic disease in 60%–80% of individuals [15]. Constant hepatic inflammation, which results in cirrhosis and hepatic fibrosis, is a characteristic of CHC [19]. However, the whole molecular mechanism of HCC pathology is still uncertain. miRNAs are small ncRNAs that can control the mRNA expression of several genes and have appeared as the main players in different biological mechanisms, including cell differentiation, division, and development. Current studies have revealed miRNAs’ participation in tumor development, as they can act as oncogenes or tumor suppressor genes [66].

MiR‐182 belongs to the miR‐183 family and is found on chromosome 7q31‐34 [27]. It was reported that miR‐182 is involved in HCV replication and pathogenesis [26]. Several research studies have described that the miR‐182 can act as both an oncomiRNA and a tumor suppressor miRNA, representing its dual role in cancer. miR‐182 dysregulation is involved in different diseases [27], like HCC [28], (NSCLC) [31], prostate cancer [29], (GBM) [32], and breast cancer [30].

CircRNAs also play a vital role in the development of HCC and other diseases [67]. CircRNAs act as ceRNA by binding with miRNAs and regulating their expression. Through the ceRNA pathway, they are vital for the growth and spread of different malignancies. CircRNAs (acting as ceRNAs) have been shown to mediate pathogenic pathways in the development of HCC, although there are still many unidentified circRNAs and their related genes that need to be studied [22].

Hsa_circ_0004812 is highly expressed in HCC patients with CHB infection, and hsa_circ_0004812 knockdown increased interferon (IFN)‐α/β production, which subsequently blocked viral multiplication and propagation [68]. HCV infection was significantly moderated after the reduction of circEXOSC and circTIAL expression in HCV‐infected cells. Moreover, it has been reported that circPSD3 is upregulated in response to HCV infection [69].

In the present study, we screened out studies related to hsa_circ_0070269, circ‐102, and circ‐166 reported in HCC through bioinformatic analysis and data mining. We also screened out all possible research papers that reported the miR‐182 role in various diseases and HCC development through literature mining. Moreover, we screened out all possible studies based on miR‐182 interactions with hsa_circ_0070269, circ‐102, and circ‐166 in HCC development. We find out all possible miR‐182 target genes, which are important key players in oncogenic pathways involved in different pathological conditions as well as in HCC development. In addition, we find different expression analysis of miR‐182, hsa_circ_0070269, and circ‐102,166 by literature mining. To validate the literature mining, we performed expression profiling analysis of miR‐182, hsa_circ_0070269, and circ‐102,166 in HCV induced HCC patients using RT‐PCR. Moreover, we found that miR‐182 was upregulated (**p < 0.0029) in HCV‐HCC patients as compared to controls, as mentioned in Figure 7A. We found out that hsa_circ‐0070269 expression was significantly low (***p < 0.002) in HCV‐HCC patients as compared to healthy individuals, as mentioned in Figure 7B. We observed that circ‐102,166 expression was significantly low (***p < 0.002) in HCV‐HCC patients as compared to controls, as displayed in Figure 7C. Furthermore, we compared the relative expression levels of miR‐182 and hsa_circ_0070269 from blood samples of HCV‐HCC patients and healthy controls. We found that hsa_circ‐0070269 was significantly downregulated and miR‐182 was highly expressed (***p < 0.0001) in the HCV induced HCC patients. As displayed in Figure 8A. In the relative expression comparison of miR‐182 and circ‐102,166, we discover that the HCV induced patients have downregulated expression of circ‐102,166 and elevated expression of miR‐182 (***p < 0.0008), as presented in Figure 8B.

It has been reported that miR‐182 could act as both an oncomiRNA and a tumor suppressor miRNA in different types of cancer [39]. MiR‐182 acts as a tumor suppressor, and downregulation inhibits cell proliferation and induces apoptosis in cervical cancer and OS [39]. MiR‐182‐5p acts as an oncogene that is highly expressed and involved in HCC progression. The circ_0003570 regulates STARD13 sponge miR‐182‐5p expression and inhibits HCC progression [40]. Lei et al. (2014) reported that miR‐182 significantly upregulates Ras homolog family member A (Rho A), which induces metastasis in breast cancer [30]. In prostate cancer, miR‐182 high expression may contribute to EMT by targeting melanocyte‐inducing transcription factor (MITF) and promoting cancer progression [70]. Yi et al. (2024) reported that circMYBL2 promotes cell proliferation and migration by sponging miR‐1205 and upregulating the E2F transcription factor 1 (E2F1) expression in HCC [71]. Circ0097009 is involved in HCC progression by sponging miR‐1261 and regulating the Solute carrier family 7 membrane 11 (SLC7A11) expression [72]. Wu et al. (2020) reported that circ‐ITCH can sponge miR‐224‐5p expression, and upregulated miR‐224‐5p targets MAF BZIP transcription Factor F (MafF) in HCC [73]. Su and colleagues observed that miR‐182 acts as an oncomiRNA in the progression of HCC by targeting NPTX1. Hsa_circ_0070269 can inhibit the cell growth and invasion of HCC by suppressing miR‐182 expression and increasing the expression of NPTX1 [74]. HCC patients have low circ‐102,166 expression. Li et al. (2021) reported that circ‐102,166 sponge the miR‐182 expression by acting as a tumor suppressor, and reduces the cell growth, migration, invasion, and tumorigenicity of HCC by upregulating the miR‐182 targets’ MTSS1 and FOXO3a [42].

In the present study, we documented the co‐expression and the interaction of miR‐182 with hsa_circ_0070269 and miR‐182 with circ‐102,166 in HCV‐induced HCC patients, in contrast to all prior studies that compared the expression of miR‐182 with hsa_circ_0070269; and miR‐182 with circ‐102,166 in HCC, and not a single study reported the expression analysis of miR‐182 with has_circ_0070269 and circ‐102,166 in HCV induced HCC patients. However, this study has some limitations, such as a relatively small sample size, and there is a need for further experimental validation at an advanced level to check the diagnostic potential of hsa_circ_0070269, circ‐102,166, and miR‐182 for HCV induced HCC.

Our data revealed miR‐182 acts as oncomiRNA and hsa_circ_0070269 and circ‐102,166 act tumor suppressors in HCV induced HCC patients. Hsa_ciic_0070269 and circ‐102,166 are highly expressed in healthy controls compared to HCV induced HCC patients, both can sponge miR‐182 expression by acting as tumor suppressors. In future, this study may help diagnose HCV‐HCC at an early stage and be used as a therapeutic target for HCV‐HCC treatment.

## 5 Conclusions

Our study suggests that hsa_circ_0070269 and circ‐102,166 are diagnostic biomarkers for HCV induced HCC. Hsa_circ_0070269 and circ‐102,166 both can sponge miR‐182 expression and in habits, HCV induced HCC development and can be used as the targets for HCV‐HCC treatment.

## Author Contributions

**Yasmeen Ishaq:** conceptualization, methodology, software, formal analysis, validation, investigation, visualization, writing – original draft. **Bisma Rauff:** investigation, writing – review and editing, formal analysis. **Badr Alzahrani:** software, formal analysis, visualization, funding acquisition, resources, validation. **Aqsa Ikram:** conceptualization, investigation, resources, writing – review and editing, visualization, supervision, validation. **Hasnain Javed:** investigation, validation, methodology. **Imran Abdullah:** resources, data curation. **Ghulam Mujtaba:** data curation, resources.

## Ethics Statement

This study has been conducted according to the guidelines of the Research Ethics Committee of the University of Lahore (REC‐UOL) (Ethical approval date: 7 June 2023). Informed consent has been taken from all participants before sample collection.

## Conflicts of Interest

The authors declare no conflicts of interest.

## Supporting information


**Table S1.** Role of miR‐182 in different diseases.
**Table S2.** Role of Hsa_circ_0070269 in HCC development.
**Table S3.** Role of circ‐102,166 in HCC development.

## Data Availability

The data that support the findings of this study are available from the corresponding author upon reasonable request.
