# Supplementary material for: Bioinformatics and Experimental Insights Into miR‐182, hsa_circ_0070269, and circ‐102,166 as Therapeutic Targets for HCV‐Associated HCC
Source: Cancer Rep (Hoboken). 2024 Dec 1;7(12):e70049. doi: 10.1002/cnr2.70049 (PMC11608829; doi:10.1002/cnr2.70049)
Supplement: Supplementary file 1 — Table S1. Role of miR‐182 in different diseases. Table S2. Role of Hsa_circ_0070269 in HCC development. Table S3. Role of circ‐102,166 in HCC development. [file CNR2-7-e70049-s001.docx]

**Supplementary Table 1: Role of miR-182 in different Diseases**

| miRNA | Disease | Expression level | Years | Reference |
| --- | --- | --- | --- | --- |
|  |  |  |  |  |
| miR-182-5p | coronary atherosclerosis (CAD) | Downregulation | 2020 | [1] |
| miR-182-5p | Lung Adenocarcinoma | Upregulation | 2021 | [2] |
| miR-182 | non-small cell lung cancer (NSCLC) | Upregulation | 2018 | [3] |
| miR-182-5p | ferroptosis in renal I/R injury | Upregulation | 2020 | [1] |
| miR-182 | Glioblastoma multiforme (GBM) | Downregulation | 2015 | [4] |
| miR-182 | prostate cancer | Upregulation | 2015 | [5] |
| miR-182 | Brest cancer | Upregulation | 2014 | [6] |
| miR-182 | melanoma | Upregulation | 2009 | [7] |
| miR-182 | high-grade serous ovarian carcinoma (HG-SOC) | Upregulation | 2012 | [8] |
| mi-182 | colon rectal cancer (CRC). | Upregulation | 2013 | [9] |
| miR-182 | Prostate Cancer | Upregulation | 2013 | [10] |
| mi-182 | clear cell renal cell carcinoma (ccRCC) | Downregulation | 2016 | [11] |
| miR-182 | HCC | Upregulation | 2019 | [12] |
| miR-182 | HCC | Upregulation | 2012 | [13] |
| miR-182 | HCC | Upregulation | 2015 | [14] |
| miR-182 | HCC | Upregulation | 2020 | [15] |
| miR-182 | HCC | Upregulation | 2020 | [16] |
| miR-182 | HCC | Upregulation | 2014 | [17] |
| miR-182 | HCC | Upregulation | 2015 | [5] |
| miR-182 | HCC | Upregulation | 2019 | [18] |
| miR-182 | HCC | Upregulation | 2021 | [19] |
| miR-182 | HCC | Upregulation | 2022 | [20] |
| miR-182-5p | non-small cell lung cancer | Upregulation | 2019 | [21] |
| miR-182-5p | Breast cancer | Upregulation | 2019 | [22] |
| miR-182 | Glioma | Upregulation | 2010 | [23] |
| Hsa-miR-182 | Lung cancer | Downregulation | 2010 | [24] |

**Supplementary Table 2: Role of Hsa_circ_0070269 in HCC development**

| Circ-RNA | Disease | Expression level | Year | Reference |
| --- | --- | --- | --- | --- |
| Hsa_circ_0070269 | HCC | Downregulation | 2019 | [18] |
| Hsa_circ_0070269 | HCC | Downregulation | 2022 | [20] |
| Hsa_circ_0070269 | HCC | Downregulation | 2021 | [25] |
| Hsa_circ_0070269 | HCC | Downregulation | 2020 | [15] |
| Hsa_circ_0070269 | HCC | Downregulation | 2021 | [26] |
| Hsa_circ_0070269 | HCC | Downregulation | 2021 | [27] |
| Hsa_circ_0070269 | HCC | Downregulation | 2020 | [28] |
| Hsa_circ_0070269 | HCC | Downregulation | 2020 | [29] |
| Hsa_circ_0070269 | HCC | Downregulation | 2021 | [30] |

**Supplementary Table 3: Role of circ-102,166 in HCC development**

| Circ-RNA | Disease | Expression level | Year | Reference |
| --- | --- | --- | --- | --- |
| Cric-102,166 | HCC | Downregulation | 2021 | [19] |
| Cric-102,166 | HCC | Downregulation | 2022 | [20] |

**References**

1. Ding, Chenguang, Xiaoming Ding, Jin Zheng, Bo Wang, Yang Li, Heli Xiang, Meng Dou, Yuxi Qiao, Puxun Tian, Wujun %J Cell death Xue, and disease. "Mir-182-5p and Mir-378a-3p Regulate Ferroptosis in I/R-Induced Renal Injury." 11, no. 10 (2020): 929.

2. Wu, X., W. Wang, G. Wu, C. Peng, and J. Liu. "Mir-182-5p Serves as an Oncogene in Lung Adenocarcinoma through Binding to Stard13." *Comput Math Methods Med* 2021 (2021): 7074343.

3. Chang, H., Y. H. Liu, L. L. Wang, J. Wang, Z. H. Zhao, J. F. Qu, and S. F. Wang. "Mir-182 Promotes Cell Proliferation by Suppressing Fbxw7 and Fbxw11 in Non-Small Cell Lung Cancer." *Am J Transl Res* 10, no. 4 (2018): 1131-42.

4. Kouri, Fotini M, Lisa A Hurley, Weston L Daniel, Emily S Day, Youjia Hua, Liangliang Hao, Chian-Yu Peng, Timothy J Merkel, Markus A Queisser, Carissa %J Genes Ritner, and development. "Mir-182 Integrates Apoptosis, Growth, and Differentiation Programs in Glioblastoma." 29, no. 7 (2015): 732-45.

5. Li, Yan, Duo Zhang, Xiaoyun Wang, Xuan Yao, Cheng Ye, Shengjie Zhang, Hui Wang, Cunjie Chang, Hongfeng Xia, Yu-cheng Wang, Jing Fang, Jun Yan, and Hao Ying. "Hypoxia-Inducible Mir-182 Enhances Hif1α Signaling Via Targeting Phd2 and Fih1 in Prostate Cancer." *Scientific Reports* 5, no. 1 (2015): 12495.

6. Lei, R., J. Tang, X. Zhuang, R. Deng, G. Li, J. Yu, Y. Liang, J. Xiao, H. Y. Wang, Q. Yang, and G. Hu. "Suppression of Mim by Microrna-182 Activates Rhoa and Promotes Breast Cancer Metastasis." *Oncogene* 33, no. 10 (2014): 1287-96.

7. Segura, M. F., D. Hanniford, S. Menendez, L. Reavie, X. Zou, S. Alvarez-Diaz, J. Zakrzewski, E. Blochin, A. Rose, D. Bogunovic, D. Polsky, J. Wei, P. Lee, I. Belitskaya-Levy, N. Bhardwaj, I. Osman, and E. Hernando. "Aberrant Mir-182 Expression Promotes Melanoma Metastasis by Repressing Foxo3 and Microphthalmia-Associated Transcription Factor." *Proc Natl Acad Sci U S A* 106, no. 6 (2009): 1814-9.

8. Liu, Z., J. Liu, M. F. Segura, C. Shao, P. Lee, Y. Gong, E. Hernando, and J. J. Wei. "Mir-182 Overexpression in Tumourigenesis of High-Grade Serous Ovarian Carcinoma." *J Pathol* 228, no. 2 (2012): 204-15.

9. Amodeo, V., V. Bazan, D. Fanale, L. Insalaco, S. Caruso, G. Cicero, G. Bronte, C. Rolfo, D. Santini, and A. Russo. "Effects of Anti-Mir-182 on Tsp-1 Expression in Human Colon Cancer Cells: There Is a Sense in Antisense?" *Expert Opin Ther Targets* 17, no. 11 (2013): 1249-61.

10. Liu, Ranlu, Jing Li, Zhigang Teng, Zhihong Zhang, and Yong %J PloS one Xu. "Overexpressed Microrna-182 Promotes Proliferation and Invasion in Prostate Cancer Pc-3 Cells by Down-Regulating N-Myc Downstream Regulated Gene 1 (Ndrg1)." 8, no. 7 (2013): e68982.

11. Wang, X, H Li, L Cui, J Feng, and Q %J Neoplasma Fan. "Microrna-182 Suppresses Clear Cell Renal Cell Carcinoma Migration and Invasion by Targeting Igf1r." 63, no. 5 (2016): 717-25.

12. Chen, Fei, Yuhong Li, Meijun Li, and Liang %J American Journal of Cancer Research Wang. "Long Noncoding Rna Gas5 Inhibits Metastasis by Targeting Mir-182/Angptl1 in Hepatocellular Carcinoma." 9, no. 1 (2019): 108.

13. Wang, Jian, Jingwu Li, Junling Shen, Chen Wang, Lili Yang, and Xinwei %J BMC cancer Zhang. "Microrna-182 Downregulates Metastasis Suppressor 1 and Contributes to Metastasis of Hepatocellular Carcinoma." 12, no. 1 (2012): 1-10.

14. Du, Chengli, Xiaoyu Weng, Wendi Hu, Zhen Lv, Heng Xiao, Chaofeng Ding, Owusu-anash K Gyabaah, Haiyang Xie, Lin Zhou, Jian %J Journal of Experimental Wu, and Clinical Cancer Research. "Hypoxia-Inducible Mir-182 Promotes Angiogenesis by Targeting Rasa1 in Hepatocellular Carcinoma." 34 (2015): 1-9.

15. Ding, Zhenghua, Li Guo, Zhongming Deng, and Peng %J Annals of Hepatology Li. "Circ-Prmt5 Enhances the Proliferation, Migration and Glycolysis of Hepatoma Cells by Targeting Mir-188-5p/Hk2 Axis." 19, no. 3 (2020): 269-79.

16. Wang, Tong‐Hong, Chau‐Ting Yeh, Jar‐Yi Ho, Kwai‐Fong Ng, and Tse‐Ching %J Molecular carcinogenesis Chen. "Oncomir Mir‐96 and Mir‐182 Promote Cell Proliferation and Invasion through Targeting Ephrina5 in Hepatocellular Carcinoma." 55, no. 4 (2016): 366-75.

17. Wang, Chenggang, Ren Ren, Haolin Hu, Changjun Tan, Miao Han, Xiaolin Wang, and Yun %J Chinese journal of cancer research Zheng. "Mir-182 Is up-Regulated and Targeting Cebpa in Hepatocellular Carcinoma." 26, no. 1 (2014): 17.

18. Chen, Z-B, W-L Cao, K Su, M Mao, X-Y Zeng, J-H %J European Review for Medical Li, and Pharmacological Sciences. "Mir22hg Inhibits Cell Growth, Migration and Invasion through Regulating the Mir-24-3p/P27kip1 Axis in Thyroid Papillary Carcinomas." 23, no. 13 (2019).

19. Olotu, F. A., and M. E. S. Soliman. "Immunoinformatics Prediction of Potential B-Cell and T-Cell Epitopes as Effective Vaccine Candidates for Eliciting Immunogenic Responses against Epstein-Barr Virus." *Biomed J* 44, no. 3 (2021): 317-37.

20. Ishaq, Yasmeen, Aqsa Ikram, Badr Alzahrani, and Sana %J Genes Khurshid. "The Role of Mirnas, Circrnas and Their Interactions in Development and Progression of Hepatocellular Carcinoma: An Insilico Approach." 14, no. 1 (2022): 13.

21. Li, Xiaoping, Bo Yang, Haixia Ren, Ting Xiao, Liang Zhang, Lei Li, Mingjiang Li, Xuhui Wang, Honggang Zhou, Weidong %J Cell death Zhang, and disease. "Hsa_Circ_0002483 Inhibited the Progression and Enhanced the Taxol Sensitivity of Non-Small Cell Lung Cancer by Targeting Mir-182-5p." 10, no. 12 (2019): 953.

22. Sang, Yuting, Bing Chen, Xiaojin Song, Yaming Li, Yiran Liang, Dianwen Han, Ning Zhang, Hanwen Zhang, Ying Liu, and Tong %J Molecular Therapy Chen. "Circrna_0025202 Regulates Tamoxifen Sensitivity and Tumor Progression Via Regulating the Mir-182-5p/Foxo3a Axis in Breast Cancer." 27, no. 9 (2019): 1638-52.

23. Jiang, Lili, Pu Mao, Libing Song, Jueheng Wu, Jieting Huang, Chuyong Lin, Jie Yuan, Lianghu Qu, Shi-Yuan Cheng, and Jun %J The American journal of pathology Li. "Mir-182 as a Prognostic Marker for Glioma Progression and Patient Survival." 177, no. 1 (2010): 29-38.

24. Sun, Yihua, Rong Fang, Chenguang Li, Li Li, Fei Li, Xiaolei Ye, Haiquan %J Biochemical Chen, and biophysical research communications. "Hsa-Mir-182 Suppresses Lung Tumorigenesis through Down Regulation of Rgs17 Expression in Vitro." 396, no. 2 (2010): 501-07.

25. Chen, Zhi, Xiang Cao, Qinyue Lu, Jingpeng Zhou, Yuhao Wang, Yanni Wu, Yongjiang Mao, Huifen Xu, Zhangping %J Food Yang, and function. "Circ01592 Regulates Unsaturated Fatty Acid Metabolism through Adsorbing Mir-218 in Bovine Mammary Epithelial Cells." 12, no. 23 (2021): 12047-58.

26. Zhou, Minghui, Puwen Zhang, Yulin Zhao, Rui Liu, and Yujie %J Journal of Cancer Zhang. "Overexpressed Circranbp17 Acts as an Oncogene to Facilitate Nasopharyngeal Carcinoma Via the Mir-635/Runx2 Axis." 12, no. 14 (2021): 4322.

27. Yan, Jia-Sheng, Qi Chen, Ya-Lin Li, and Yun-qiu %J Cell Cycle Gao. "Hsa_Circ_0065217 Promotes Growth and Metastasis of Renal Cancer through Regulating the Mir-214-3p-Alpk2 Axis." 20, no. 23 (2021): 2519-30.

28. Zhang, Meng, Li Ma, Yuhan Liu, Yonglong He, Guang Li, Xiaopeng An, and Binyun %J Genes Cao. "Circrna-006258 Sponge-Adsorbs Mir-574-5p to Regulate Cell Growth and Milk Synthesis Via Evi5l in Goat Mammary Epithelial Cells." 11, no. 7 (2020): 718.

29. Yang, Tian Bao, Fang Yi, Wei Feng Liu, Yan Hui Yang, Cheng Yang, and Junjun %J Journal of Cancer Sun. "Identification of Hsa_Circ_0039053 as an up-Regulated and Oncogenic Circrna in Hepatocellular Carcinoma Via the Mir-637-Mediated Usp21 Activation." 11, no. 23 (2020): 6950.

30. Wu, Shanshan, Shimei Liu, Huaihua Song, and Jiayu %J Journal of Investigative Medicine Xia. "Circular Rna Hipk3 Plays a Carcinogenic Role in Cervical Cancer Progression Via Regulating Mir-485-3p/Fgf2 Axis." 69, no. 3 (2021): 768-74.
